# New Breakthroughs in the Diagnosis of Leptomeningeal Carcinomatosis: A Review of Liquid Biopsies of Cerebrospinal Fluid

**DOI:** 10.7759/cureus.55187

**Published:** 2024-02-28

**Authors:** Maria Goldberg, Michel G Mondragon-Soto, Ghaith Altawalbeh, Bernhard Meyer, Amir Kaywan Aftahy

**Affiliations:** 1 Department of Neurosurgery, School of Medicine, Klinikum Rechts der Isar, Technical University of Munich, Munich, DEU; 2 Neurological Surgery, Instituto Nacional de Neurologia y Neurocirugia, Mexico, MEX

**Keywords:** brain spine tumors, neuro oncology, brain metastases, leptomeningeal carcinomatosis, liquid biopsy

## Abstract

Leptomeningeal carcinomatosis represents a terminal stage and is a devastating complication of cancer. Despite its high incidence, current diagnostic methods fail to accurately detect this condition in a timely manner. This failure to diagnose leads to the refusal of treatment and the absence of clinical trials, hampering the development of new therapy strategies. The use of liquid biopsy is revolutionizing the field of diagnostic oncology. The dynamic and non-invasive detection of tumor markers has enormous potential in cancer diagnostics and treatment. Leptomeningeal carcinomatosis is a condition where invasive tissue biopsy is not part of the routine diagnostic analysis, making liquid biopsy an essential diagnostic tool. Several elements in cerebrospinal fluid (CSF) have been investigated as potential targets of liquid biopsy, including free circulating tumor cells, free circulating nucleic acids, proteins, exosomes, and even non-tumor cells as part of the dynamic tumor microenvironment. This review aims to summarize current breakthroughs in the research on liquid biopsy, including the latest breakthroughs in the identification of tumor cells and nucleic acids, and give an overview of future directions in the diagnosis of leptomeningeal carcinomatosis.

## Introduction and background

Leptomeningeal carcinomatosis (LC) is the metastatic dissemination of cancer cells, mostly originating from breast, lung, and melanoma cancer, to the pia mater, arachnoid, and/or subarachnoid space. It is also known as leptomeningeal metastasis, carcinomatous meningitis, or leptomeningeal disease (LMD) [1,2]. Metastatic cells present in the cerebrospinal fluid (CSF) may invade the central nervous system over a short timeframe, disrupting the brain and spinal cord; on occasion, this can cause sudden neurological deterioration and death. Neoplastic cells may reach the brain through a blood vessel and migrate from the choroid plexus to the CSF. Once they enter the leptomeninges, metastatic cancer cells can circulate with freedom, causing extensive damage to the central nervous system [3]. The current review will highlight the diagnostic frontiers regarding LC and provide a broad panorama of the actual diagnostic methods to detect and monitor disease in the CSF, as well as discuss possible future directions of research on this disease. 

Epidemiology and pathophysiology 

The true incidence of LC remains unclear, as it ranges from 8% to 35% across studies. The reasons for this broad range are multifactorial, including the tumor histology, location, and size; pial involvement; and the type of surgical resection [4]. Up to 15% of patients with solid tumors are diagnosed with LC, and similar rates are observed in patients with leukemia and lymphoma [5-8]. 

Resection has become an essential treatment modality for newly diagnosed brain metastases. Nonetheless, studies have suggested an association with a higher risk of developing LC. The latter is due to the disruption of anatomical barriers in the brain tissue and the surgical spillage of tumor cells, resulting in the dissemination of tumor cells. 

In a meta-analysis performed by Twarie and collaborators, among 386 patients, 18 risk factors were reported as significantly positively associated with LC occurrence, including, but not limited to, a larger tumor size, infratentorial location of bone metastases (BMs), proximity of BM to cerebrospinal fluid spaces, ventricle violation during surgery, subtotal or piecemeal resection, and postoperative stereotactic radiosurgery. In breast cancer, lymph node metastasis of the primary tumor (HR = 2.73, 95% CI: 2.12-3.52) and multiple BMs (HR = 1.37, 95% CI: 1.18-1.58) were significantly associated with a higher risk of LC occurrence after neurosurgery [9]. 

The development of LC is thought to be caused by CSF seeding through the leptomeninges by hematogenic, perineural, or direct tumor growth. Thus, an examination of CSF may reveal potentially useful biomarkers for the diagnosis and/or prognosis of LC development.

Current diagnostic workup 

Clinical Manifestations

Patients with LC usually present with an existing history of malignancy as well as with a known metastatic disease. A small fraction of the patients has a synchronous or exclusive initial presentation of LC. The great majority of the patients are symptomatic, and these symptoms may be either localizable, due to the involvement of specific neuroanatomic structures, or diffuse, which commonly occurs secondary to the increased intracranial pressure that is frequently associated with communicating hydrocephalus. Localizable symptoms are often multifocal due to the broad involvement of the surface of the brain, spinal cord parenchyma, or other structures that traverse the CSF. This can manifest as multiple cranial neuropathies, especially the involvement of cranial nerves VI, VII, and VIII, or radiculopathies. The chronological presentation is often stable before experiencing an abrupt clinical decline. The natural history of LC is not well delineated, but the evolution from the presentation of the symptoms to death is rather short in most cases [10]. Although LC usually presents as the terminal stage of widely disseminated systemic cancers, up to 10% of patients present with LC as the first cancer manifestation [11]. 

Diagnostic Evaluation

LC diagnosis is often made through a combination of physical examination, neuroimaging, clinical suspicion, and CSF analysis [10], and the presence of this pathology should be suspected and evaluated more frequently, particularly if there is a known history of malignancy. 

**Figure 1:**
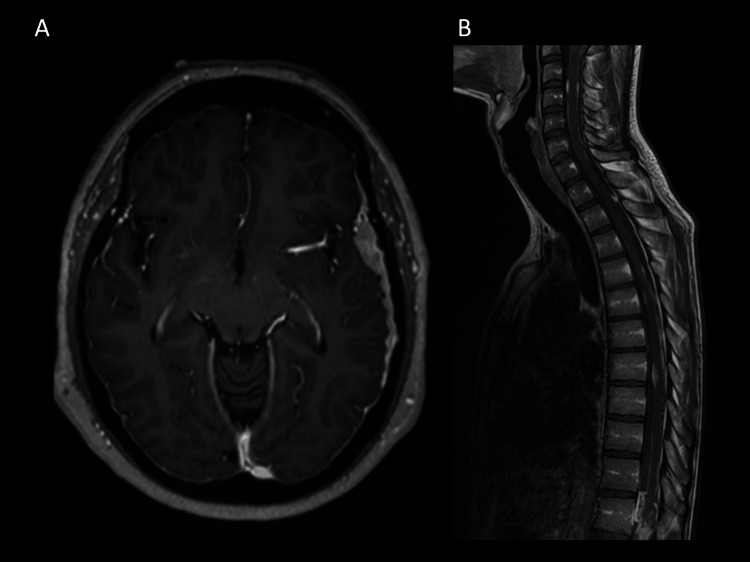
Example of LMD with T1 contrast-enhanced lesions in brain (A) and spine (B). LMD: leptomeningeal disease. Note: This image is the author's own creation.

The imaging modality of choice for evaluating LC is magnetic resonance imaging (MRI) with contrast [2,9,12,13]. An example of LMD in the brain and spine can be seen in Figure 1. In previous reports, radiographic signs have been reported to be associated with LC that include two different patterns: classic sugar-coating (cLC) and nodular (nLC). The radiographic pattern of LC has been previously demonstrated to be prognostic, with cLC having significantly worse overall survival (OS) compared with nLC [14,15].

Classical leptomeningeal disease (cLMD) includes an enhancement in the cranial nerves, cisterns, cerebellar folia, and sulci [16]; Zuckerguss or diffuse “sugar-coating” enhancement is also observed across the surface of the brain [4,17-19].

The proposed definition of nLC is the presence of a focal nodule or nodules adherent to surfaces with CSF contact, including the dural or pial surface, tentorium, ventricles, and hypervascular dural tail [4].

The downside of the MRI is its limited sensitivity; however, this is secondary to an incomplete understanding of the sensitivity and specificity of other diagnostic modalities, including the best currently available diagnostic modality, i.e., CSF cytology. The MRI protocol should include a contrasted spinal MRI of melanoma and lung cancer who are highly suspected of having CNS metastases. 

Other issues concerning the radiological diagnosis also involve discrepancies in the literature in how the radiological findings are defined. Several efforts have been made to unify the different definitions proposed, as well as to be consistent with the Response Assessment in Neuro-Oncology (RANO) criteria for metastases [4,19].

CSF cytologic assessment is currently thought to be the best definitive diagnostic modality. It has a high specificity of up to 100% with a low false-positive rate, although with limited sensitivity with a maximum of 75% [17,20]. The preferred method for collecting CSF for diagnostic purposes is through a lumbar puncture [10,13,20]. However, lumbar punctures should only be performed after proper neuroimaging to avoid performing the procedure in a patient at risk from herniation due to major brain metastases or complications from local bulky disease [21]. It is also possible to encounter unspecific abnormalities during the lumbar puncture; for example, increased opening pressure has been detected in 21-24% of patients [22-24], increased leukocyte count in 39-77.5%, elevated protein in 56-91%, and decreased glucose in 22-38% of patients [24-26] (Table 1).

**Table 1:** CSF normal reference values in the cytologic assessment. CSF: cerebrospinal fluid.

Finding	Values
Opening pressure	>200 mm H_2_O
Leukocyte count	>4 per mm^3^
Protein	>500 mg/L
Glucose	<600 mg/L

Whenever lumbar puncture is not possible, an imaging-guided cisternal puncture should be considered. The current recommendation is to perform the analysis on a large volume (optimally >10 mL, with a minimum of 5 ml) that has been freshly obtained (collected less than 30 minutes before the analysis), accompanied by immunohistochemical staining for epithelial and melanocytic markers to optimize the diagnostic accuracy of the test [10,13]. However, the sensitivity may be affected by the location from which the CSF is obtained (lumbar vs. ventricular) [20].

Tumor cells can be detected at diagnosis in up to 80% of LC cases. The CSF cytologic analysis should be reported as positive when the presence of malignant cells in the CSF is observed; equivocal when detecting “suspicious” or “atypical” cells in the CSF; or negative when no malignant or potentially malignant cells are found in the CSF [21].

Thus, a diagnosis of LC can be confirmed in the presence of tumor cells in the CSF or by a positive leptomeningeal biopsy. In the absence of a confirmed diagnosis, the diagnosis of LC can be considered as probable in a patient with a history of histologically proven cancer with a reasonable risk of LC and after discarding other differential diagnoses. The diagnosis is probable when typical clinical findings or typical MRI findings are present without a confirmed CSF test [21].

Genetic Mutations in Leptomeningeal Carcinomatosis

LC is a life-threatening condition that is associated with a poor functional and life prognosis, a consequence often seen with solid tumors, regardless of treatment [27]. Although its current incidence is low, the clinical impact of LC on the patients and its increasing incidence makes the identification of biomarkers and exact molecular mechanisms a priority for the prognosis and the treatment of LMD [3]. 

The molecular mechanism underlying LC is not yet fully understood. A meta-analysis conducted by Congur et al., which included 16 countries, identified five different genes as the most commonly mutated genes in all three types of cancer included in the study (non-small-cell lung cancer, breast cancer, and melanoma). The genes were TP 53, PTEN, PIK3CA, IL7R, and KMT2D. These genes are associated with the regulation of cell communication and signaling and cell proliferation [3].

Signaling pathways are responsible for cell-to-cell communication, cell turnover, death, and cellular movement. The PI3K-AKT and RAS-ERK pathways regulate processes like cell proliferation and cell-to-cell communication. Alterations in any of the aforementioned genes can therefore affect the signaling pathways controlling cancer progression, causing cells to profusely proliferate [3]. On the other hand, in patients with breast cancer, the HER-2 mutation has been found to decrease the risk of LC development [9].

Unfortunately, as stated before, the prognosis for patients with LC remains significantly poor. Unlike the diagnostic-specific graded prognostic assessment (DS-GPA) [28], there are currently no such strong prognostication systems [28] for LC [10].

## Review

Application of liquid biopsies

A better understanding of cancer biology, dynamic system modulation, and cell interaction indicates the importance of approaching cancer as a systemic disease [29]. Moreover, the need for early detection and available screening methods is obvious in preventing and halting the progression of cancer [30]. Nevertheless, the gold-standard method of tumor profiling is still the local-tissue biopsy [31]. However, there is not only the problem of the invasiveness of the procedure but also the inability to dynamically monitor the tumor molecular profile since only one tissue sample can be obtained at any one time [32]. On the other hand, tumor molecular monitoring and the identification of drug resistance markers and escape mutations have drastically improved personalized therapies [33]. These factors have shifted the focus in modern oncology toward less invasive and more dynamic methods of tumor detection [34]. 

**Figure 2:**
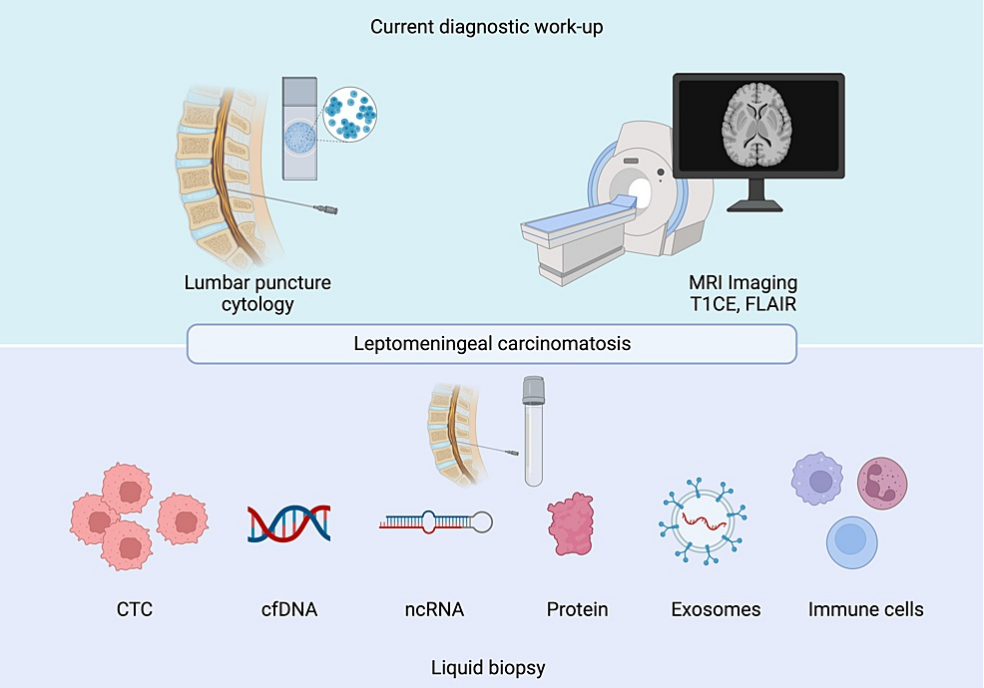
Methods to diagnose leptomeningeal carcinomatosis. Note: This image is created by Biorender.com.

Liquid biopsy (LB) is a concept that was introduced in 1993 in Hamburg [35]. As the name implies, LB is a method for the testing of tumor markers in bodily fluids, such as blood, urine, saliva, and CSF [36]. LB demonstrates several advantages in comparison to traditional biopsy, including non-invasiveness, continuous tumor monitoring, higher sensitivity, and cost-effectiveness [37]. It is well known that tumors release various substances into the circulatory system, which can further migrate throughout the body and reach distant organs [38]. These substances include circulating intact tumor cells, tumor-released nucleic acids, proteins, and tumor-derived extracellular vesicles or exosomes, as well as rearranged immune cells, allowing multiple techniques to be used to analyze these varied groups of structures (Figure 2) [37]. LB appears promising in the diagnosis of LM, demonstrating superiority in terms of accuracy over standard diagnostic methods [39]. 

Circulating tumor cells (CTCs)

Discovered in 1869 [40], free circulating tumor cells (CTCs) have become a valuable tool in cancer detection via LB [41]. Not only can CTCs be detected in small fluid volumes [42], but they may also be cultured in vitro for more sophisticated analyses [43]. The number of CTCs in the blood was shown to correlate with increased metastatic burden and overall survival [41,44,45]. CTCs can be detected in the blood in 70% of cases of cancer metastases [46]. The most-used technique, and the only FDA-approved method, is the CellSearch® assay, which examines epithelial cell adhesion molecule (EpCAM) expression, nuclear staining with 4′, 6-diamidino-2-phenylindole (DAPI), and the immunofluorescence detection of cytokeratin and CD45 [47]. 

The CellSearch® assay (Veridex) consists of a semiautomated system for the preparation of a sample that is enriched for cells expressing EpCAM using antibody-coated magnetic beads. The cells are labeled with the fluorescent nucleic acid dye DAPI, and fluorescently labeled monoclonal antibodies specific for leukocytes (CD45-allophycocyanin) and epithelial cells (cytokeratin 8,18,19-phycoerythrin) are used to distinguish the cells [47]. This test also allows for the additional analysis of specific markers, such as HER2 expression [48], which have proven to be accurate in detecting metastatic cancer.

The number of CTCs strongly correlates with overall survival and tumor progression [41,49-51]. There was an attempt to detect CTCs in the peripheral blood of patients with LM; however, their concentration was significantly lower in comparison to CSF [52], or they could not be detected at all [53]. CSF provides nourishment and waste removal, making it a unique diagnostic material in the investigation of diseases of the central nervous system [54]. Metastatic cells are believed to infiltrate the CSF through Virchow-Robin spaces, the lymphatic system, or via direct infiltration of parenchymal metastases [55-57]. CSF shows low background levels of cell-free DNA, protein, and low lipid content, making it an ideal substance for LC diagnosis [41]. A recent meta-analysis reported a cumulative sensitivity and specificity of the method of 87% and 94%, respectively [58]. A summary of CTC studies in LM research is presented in Table 2. 

**Table 2:** CTCs detection research in CSF in patients with LC. CTCs: circulating tumor cells; CSF: cerebrospinal fluid; LC: leptomeningeal carcinomatosis; DAPI: 4′,6-diamidino-2-phenylindole.

Marker	Technique	Disease	Number of samples	Clinical benefit	Year	Reference
EPCAM-PE, DAPI, CD45	CellSearch® assay	Breast cancer	5	Diagnostics, treatment response, tumor burden; number of CTCs correlates with functional status	2011	[59]
EpCAM, DAPI, Cytokeratin 8/8/19, CD45	Adapted CellSearch® assay	Breast cancer	8	Diagnostics, treatment response	2012	[60]
EpCAM	Flow cytometry	Epithelial-cell tumors	78	Sensitivity 76%, specificity 96%	2012	[61]
CD146, HMW/MAA, CD34, CD45, DAPI	CellSearch® assay	Melanoma	2	Quantitative diagnostic	2013	[53]
EpCAM, DAPI, Cytokeratin 8/8/19, CD45	CellSearch® assay	Lung cancer, breast cancer	51	Sensitivity 100%, specificity 97%	2013	[62]
EpCAM, DAPI, Cytokeratin 8/8/19, CD45	CellSearch® assay	Lung cancer	18	Sensitivity 78%, specificity 100%	2015	[63]
EpCAM, DAPI, Cytokeratin 8/8/19, CD45	CellSearch® assay	Breast cancer	38	Sensitivity 81%, specificity 85%	2015	[64]
EpCAM, Hoechst, CD45-	Flow-cytometry	Breast cancer, lung cancer, ovarian cancer	29	Sensitivity 100%, specificity 100%	2016	[65]
CD326, CD45, CD33	Flow-cytometry	-	6	Sensitivity 97%, specificity 99%	2016	[66]
EpCAM, DAPI, Cytokeratin 8/8/19, CD45	CellSearch® assay	Lung cancer	21	Sensitivity 95%, specificity 100%	2017	[67]
EpCAM, DAPI, Cytokeratin 8/8/19, CD45	CellSearch® assay	Lung cancer, breast cancer, miscellaneous	95	Sensitivity 93%, specificity 95%	2017	[68]
DAPI/CD45/CK	SEi FISH	Breast cancer	8	Monitoring of intrathecal therapy	2018	[69]
EpCAM, DAPI, Cytokeratin 8/8/19, CD45, CD34, CD146, HMW/MAA	CellSearch® assay	Breast, lung, renal cell cancer, melanoma	20	Sensitivity 89%, specificity 100%	2020	[70]
EpCAM	Flow-cytometry	Breast cancer, lung cancer, ovarian cancer	72	Sensitivity 94%, specificity 100%	2020	[71]
EpCAM, HER2	CellSearch® assay	Epithelial HER2+ cancers	34	Quantification, tumor progression, treatment response	2020	[72]
EpCAM, DAPI, Cytokeratin 8/8/19, CD45	CellSearch® assay	Lung cancer, breast cancer, gastrointestinal cancer	101	Prediction of survival	2022	[73]
EpCAM, DAPI, Cytokeratin 8/8/19, CD45, HER2	CellSearch® assay	Breast cancer	49	Sensitivity 100%, specificity 77%	2022	[74]
Antibody cocktail + ER/HER2	CNSide®	Breast cancer	10	Sensitivity 100%, specificity 83%	2022	[75]

The identification and quantification of CTCs in CSF is a promising diagnostic approach that has the potential to assess therapy response and dynamic monitoring in LC treatment. It shows superiority over classical cytology staining methods and is becoming more accessible in the clinical routine [57]. Some cells, however, are known to lose EpCAM expression, making these methods unsuitable for LC diagnosis [76]. Not only can cells with low or no EpCAM expression not be detected, but they are also either dead or immobilized at the moment of their capture, making further analysis complicated [77]. Additionally, there is a certain level of heterogeneity within this population of cells [78]. 

cfDNA

Dying tumor cells, or cells undergoing apoptosis, release tumor-cell DNA or cell-free DNA (cfDNA) into the circulatory system [79]. Advances in modern technology allow for the analysis of these fragments via the investigation of mutations, including single nucleotide variants (SNVs), deletions and insertions, gene fusion, copy number variations (CNVs), and large chromosomal rearrangements [80-82]. It is now known that the brain metastasis (BM) genetic profile differs from the one in the primary tumor, recurrent tumor, and even between different metastatic lesions, which makes it essential to analyze the actual metastatic cells themselves [83]. The detection of mutations in real-time allows for the analysis of therapy resistance mechanisms and appropriate clinical decision-making [84,85]. CSF enriched with cfDNA shows a low background of genomic DNA in comparison to blood samples, which, as mentioned above, makes it an optimal resource for sophisticated and precise mutation analysis, and it may be even more sensitive in the case of parenchymal BM [57,86]. Due to the blood-brain barrier, there is a very low enrichment of cfDNA in plasma [87]. It has been shown that CSF is relatively stable at -80°C [88]. Moreover, it more reliably identifies driver mutations compared with plasma [89,90]. Not only are they useful as diagnostic markers, but escape mutations can be detected dynamically before disease progression [91]. The published articles on cfDNA and LM are summarized in Table 3.

**Table 3:** cfDNA detection research using CSF in patients with LC. CSF: cerebrospinal fluid; LC: leptomeningeal carcinomatosis; cfDNA: cell-free DNA; EGFR: epidermal growth factor receptor; CTC: circulating tumor cells.

Marker	Technique	Disease	Number of samples	Clinical benefit	Year	Reference
8q24 gain (from CTC)	Array CGH	Breast cancer	15	Early diagnosis, targeted therapy	2013	[92]
TP53, EGFR, MLL2, NSD1, CREB3L1, TPR, TSC2	ddPCR	Breast cancer	1	Targeted detection of driver mutations	2015	[93]
BRAFV600E/K	ddPCR	Melanoma	11	Quantification, therapy-response monitoring	2016	[94]
MET amplification, ERBB2 mutation	NGS	Non-small cell lung cancer (NSCLC)	19	Diagnostics	2017	[67]
BRAF600E, NRAS, PIK3CA, ABL1, MET	ddPCR, NGS	Melanoma	7	Diagnostics	2018	[95]
MET/ERBB2/KRAS/ALK/MYC CNV, TP53 LOH, EGFRT790M	NGS	EGFR mutant lung cancer	26	Genetic profile of CSF cfDNA, diagnosis, marker of disease progression	2018	[89]
Mutation rate in 168 lung cancer-related genes, MaxAF, TP53 LOH, EGFR mutations, ALK fusions, and ERBB2 amplification	Capture-based targeted sequencing	Lung cancer	72	CSF as a better sample for liquid biopsy than blood	2018	[96]
EGFR	ddPCR	Lung cancer	15	EGFR therapy monitoring	2019	[97]
EGFR	NGS	Lung cancer	11	Targeted detection of driver mutations, not suitable for LM diagnostics	2020	[98]
EGFR, TP53	Error-suppressed deep sequencing	Lung cancer	2	Targeted detection of driver mutations, not specific for LM diagnostics	2021	[99]
EGFR C79C7S, MET ampl	Nanowire-based cfDNA	Lung cancer	11	EGFR therapy monitoring	2021	[100]
Cancer fraction using ichorCNA	NGS	-	22	Increased sensitivity and specificity in comparison to cytology	2021	[101]
Genome-wide aneuploidy	NGS, mFAST-SeqS	Breast cancer	10	LM prediction, survival prognosis	2021	[102]
EGFR mutation, T790M	ddPCR	EGFR-mutant lung cancer	48	68.8% positive for EGFR mutation, 14.6% positive for T790M, therapy monitoring	2021	[103]
TP53, PIK3CA, CCND1	NGS	Breast cancer	8	Targeted detection of driver mutations	2022	[75]
cfDNA fraction	ulpWGS	Breast cancer	30	Quantitative marker for diagnostic and treatment response	2022	[104]

The cfDNA analysis provides a unique opportunity to detect molecular markers that are not present in BM or blood [105]. More and more studies are attempting to avoid targeted marker testing and focus on broad approaches in order to identify new mutations. These would allow one to test for tumor heterogeneity better than by tissue biopsy [106]. The problem, however, is finding a marker specific to LC but not the cfDNA, which arises from coexisting metastatic lesions in the nervous system. A variation in nucleosome positioning in cfDNA was proposed as a method for identifying the tissue origin [107]. Unfortunately, the majority of the current studies are retrospective and small in sample size, limiting their application in a clinical setting. Larger prospective studies investigating broad molecular characteristics are required for the development of a valid, accurate, and reliable diagnostic method. 

RNA 

Another element that can be inspected in CSF is cell RNA. DNA mutations do not necessarily lead to downstream changes. Additionally, RNA represents a “snapshot” of genetic changes and disease progression [108]. In addition to coding RNA, long non-coding RNA and microRNA, which are regulatory non-protein-coding transcripts that regulate protein expression, have also been thoroughly investigated in the past few years as potential biomarkers [109]. Due to the low number of cancer cells in CSF and the difficulties in differentiating these from immune cells, the idea of RNA identification was proposed, since even a low concentration of RNA can be detected [110]. 

Based on the single-cell RNA-sequencing analysis of CSF in five patients with breast cancer and NSCLC, a higher expression of the iron-binding protein lipocalin-2 and its receptor, SCL22A17, was identified in cancer cells as a result of the macrophage cytokine response [90]. A trial of four CSF samples from patients with NSCLC revealed that CEACAM6 and MUC1 were present as cfRNA and that they were associated with disease progression and cell migration [111]. The transcription factor SREBF2 was also found to be activated in the CSF of five patients with breast and lung cancer.

Many physiological processes, including oncogenesis, are regulated by non-coding RNA, such as microRNA [112]. In a recent study, several microRNAs, namely miR-335-5p, miR-34b-3p, and miR-466, were identified in the CSF of 22 LC patients with lung, breast, and ovarian cancer; these differed from those found in primary and metastatic lesions, suggesting them as potential candidates as LC diagnostic markers [113]. 

Protein markers 

Proteins are being widely used in clinical practice as diagnostic markers since they reflect pathophysiological changes under many different conditions [114]. Several unsuccessful attempts have been made to analyze well-known protein biomarkers such as CA15-3 or CEA in CSF, which have proven to be non-specific when found in CSF and in the presence of any intraparenchymal lesions [115-117]. Another commonly used marker, VEGR, when standardized to serum, can be detected in the CSF of LC patients [118]. A higher expression of complement component 3 was observed in the CSF of 37 patients with confirmed LC based on ELISAs. This protein is believed to play a role in disrupting the blood-brain barrier and in cancer growth [119]. Lipocalin 2, an iron-binding protein, and its receptor were exclusively expressed in cancer cells in the CSF of five patients with breast and lung cancer LC, which was confirmed by ELISA and flow cytometry. The authors, moreover, showed inhibition of cancer growth when iron chelation therapy was applied [90]. Increased activities of matrix metalloprotease 9 and A Disintegrin and Metalloproteases (ADAMs) 8 and 17 were detected in the CSF of 12 LM patients as indicators of the breakdown of the blood-brain barrier based on a proteolytic activity matrix assay [120].

Modern research techniques have been expanded from single-protein detection, such as staining for the known protein markers HER2 and BRAF V600E [57], toward more sophisticated proteomic methods that can assess multiple proteins at the same time [121]. Proteomic-based methods are increasingly being used for the identification of CSF markers in different diseases, including LC [122,123]. Mass spectrometry analysis revealed an upregulation of the pathways enrolled in innate immunity and acute-phase response signaling in the CSF of eight LC patients with melanoma, with high-level expression of the TGF-β1 protein confirmed via ELISA [124]. Several proteins specific to lymphoma and leukemia LC, including PTPRC, SERPINC1, sCD44, sCD14, ANPEP, SPP1, FCGR1A, C9, sCD19, and sCD34, were identified in the CSF of 12 patients based on liquid chromatography-tandem mass spectrometry [125].

Tumor microenvironment

The tumor microenvironment (TME) plays an essential role in cell invasion and cancer progression. It consists of immune cells surrounded by the extracellular matrix [126,127]. One of the key components of TME is exosomes, which are lipid bilayer spheroids that contain protein and nucleic acids [128]. Moreover, they have been shown to pass through the blood-brain barrier [129]. The separation of exosomes is possible using ultracentrifugation methods, with subsequent analysis of the marker of interest [130]. The FN1 (fibronectin) exosomal protein, known to be widely expressed in different tumor cells, may serve as a potential diagnostic marker in NSCLC LC diagnosis based on the proteomic analysis of patients with confirmed LC [131]. Another recent study proposed two exosomal microRNA markers, 509-3p and 449a, in the CSF of NSCLC progression as indicators of LC [132]. Exosomal miRNA-483-5p and miRNA-342-5p were found to be significantly upregulated in both the serum and CSF of LM patients with NSCL [133]. 

It is well known that immune cells are present in the central nervous system and the CSF [134]. In a prospective study, a certain shift pattern in the immune cell presence in CSF was observed in patients with LM. CD8+ T cells were highly present after immune checkpoint inhibitor administration, and the upregulation of IFN-γ signaling was observed [134]. Research on the IFN-γ signaling response may be used in the dynamic assessment of checkpoint inhibitor therapy as an independent marker of tumor cell detection. The dominant presence of M2-polarized macrophages was observed in the CSF of patients with LM based on scRNAseq, displaying immunosuppressive characteristics and showing high levels of ligand-receptor interactions such as MDK‐SORL1 and MDK‐LRP1 [135]. Another interesting phenomenon was observed in seven LM patients with breast cancer. Based on flow cytometry analysis, there was a lower number of CD45+ and CD8+ T-cells and a higher frequency of Treg cells in CSF [136]. Thus, examining the shift in the pattern of immune cells may also be useful in the diagnosis of LC.

## Conclusions

Leptomeningeal carcinomatosis in metastatic cancer is a highly morbid and lethal entity in patients with oncologic disease. Currently, there are no effective and reliable methods for early diagnosis, which dampens the opportunity for the optimal management of this group of patients.

Several studies have sought to identify an optimal diagnostic test that is reliable, enables continued monitoring of the disease’s activity, is inexpensive, and, above all, does not significantly increase the morbidity of the already affected oncologic patients. New methods of identifying tumor cells directly or nucleic acid can not only provide an accurate method of diagnostics but also allow monitoring of mutations necessary for targeted therapies. Early detection may lead to new therapeutic approaches, which, consequently, may improve the quality of life of patients. Liquid biopsies remain, however, challenging due to ongoing method establishment and high procedural costs. While the different options seem promising, more combined efforts are still required to obtain more accurate data.
